# Which stem in total hip arthroplasty for developmental hip dysplasia? A comparative study using a 3D CT-based software for pre-operative surgical planning

**DOI:** 10.1186/s10195-022-00650-x

**Published:** 2022-07-15

**Authors:** Francesco Castagnini, Stefano Lucchini, Barbara Bordini, Monica Cosentino, Francesco Pardo, Francesco Traina

**Affiliations:** 1 Ortopedia-Traumatologia e Chirurgia protesica e dei reimpianti d’anca e di ginocchio, IRCCS Istituto Ortopedico Rizzoli, Via Pupilli 1, 40136 Bologna, Italy; 2 DIBINEM, Università di Bologna, Bologna, Italy; 3 Laboratorio di Tecnologia Medica, IRCCS Istituto Ortopedico Rizzoli, Via di Barbiano 1/10, 40136 Bologna, Italy

**Keywords:** Single wedge, Conical tapered, Combined anteversion, Offset, Antetorsion

## Abstract

**Background:**

Stem choice in total hip arthroplasty (THA) for hip dysplasia is still controversial. The aims of the study were to evaluate (1) which stem design provided the highest percentage of adequate reconstructions in THA for dysplasia and (2) any correlation between the reconstructions provided by the stems and the native femoral morphology.

**Materials and methods:**

150 CT scans including 200 adult dysplastic hips were randomly selected. Using the 3D CT-based software Hip-Op for surgical planning, the native hip anatomy was studied. Then, a single wedge tapered stem, an anatomical stem and a conical tapered stem were simulated in every hip. An adequate reconstruction of hip biomechanics was obtained when combined anteversion, offset restoration, coronal and sagittal tilt, canal filling and leg lengthening were inside the normal ranges.

**Results:**

Conical stems achieved the highest percentage of adequate reconstructions (87%, *p* < 0.0001). The anatomical stem was the worst performer. Single wedge and anatomical stem acceptability was mainly influenced by the combined anteversion. Stem anteversion was correlated with the femoral anteversion (fair correlation), the calcar femorale (fair) and the mediolateral femoral diameter at isthmus (poor). When the femoral anteversion was ≥ 25°, combined anteversion was very acceptable for the conical stem (99.2%), whereas the rate of acceptable combined anteversion for the single wedge tapered stem was 71.4%, and that for the anatomical stem was 51.6% (*p* < 0.0001).

**Conclusions:**

Stem choice in developmental hip dysplasia is mainly driven by appropriate combined anteversion, which is dependent on the coronal and axial femoral morphologies. As a rule of thumb, tapered stems are adequate when femoral anteversion is < 25°; conical stems should be adopted for higher anteversions.

**Level of evidence:**

IV.

## Introduction

The complex femoral morphology in developmental hip dysplasia (DDH) may pose several challenges to the reconstructive surgeons performing total hip arthroplasty (THA) [1–6]. In DDH, the main anatomic alterations are a high neck-shaft angle, a short offset, a narrow canal and, above all, a high femoral anteversion; these features, as well as the femoral anteversion, are not always correlated to the Crowe classification [1–3].

The wide variability and the complex geometry of dysplastic femurs make stem choice in THA critical to reconstructing hip biomechanics and confering implant stability [5]. To date, no guidelines and no thresholds based on the native morphology are currently available to facilitate a specific stem choice [4–6]. While some authors have empirically suggested conical tapered stems for femoral anteversions of 30–60° and for small femurs, the optimal stem design to use in DDH is still unknown [4–6].

Thus, we sought to determine (1) which stem design provided the highest percentage of adequate reconstructions in THA for DDH, in terms of combined anteversion, offset restoration, coronal and sagittal tilt, canal filling, and leg lengthening, and (2) any correlation between the reconstruction provided by each stem and the native femoral morphology. We hypothesized that in DDH, conical tapered stems could provide safe combined anteversion, reliable canal filling and adequate positioning in most cases, especially when the femoral anteversion is > 25°.

## Materials and methods

The study was approved by the local ethical committee and registered on ClinicalTrials.gov (NCT04904640).

The hospital database was screened for pelvis CT scans that included a native dysplastic hip and were performed during 2000–2018 for pre-operative THA planning or painful THA diagnosis, with the aim being to achieve 200 eligible hips.

The inclusion criteria were:

- Definition of DDH according to Wiberg (center edge angle < 20°) [7]
- Pre-operative CT scan extended from the fourth lumbar vertebra to the tibial plateau.

Hips with other congenital or acquired pathologies, arthroplasties or inadequate CT scans were excluded.

200 hips in 150 CTs (150 Caucasian patients) were randomly selected. The first author simulated the implantation of three different stem designs per hip using a 3D CT-based pre-operative planning software (Hip-Op) after an appropriate assessment of the native femoral morphology.

### 3D CT-based pre-operative software for surgical planning: Hip-Op

The software reproduced a 3D CT-based planning environment [8, 9]. The user-friendly graphical user interface is based on a multimodal display visualization. The user can choose the components from a library of selected implants: the implants and the patient anatomy are rendered in each view. The planner may evaluate the implant type, size and position by interactively moving and rotating the components in the view area. The validity of Hip-Op software as a pre-operative planning aid has previously been assessed [9].

### Implant features and planning technique

The planning was performed by a single arthroplasty surgeon (the first author) in every case. Three non-modular stems were selected based on their design and classification according to Khanuja et al. [10]: CLS (Zimmer Biomet, Warsaw, USA) single wedge tapered stem, type 1; Aptafix (Adler Ortho, Milan, Italy) anatomical stem, type 6; Wagner Cone (Zimmer Biomet) conical tapered stem, type 3B [11–13]. The CLS had 13 stem sizes with three CCD angles (125°, 135° and 145°) [11]. The Aptafix had a standard version and a offset solution (7.5 mm lateralization), both with a CCD angle of 135°: eight possible stem sizes are available [12]. The Wagner Cone had 12 stem sizes with two CCD angles, 125° and 135° [13]. The acetabular cup was the Continuum (Zimmer), which was adopted for every single stem simulation.

In all the dysplastic hips, the surgical planning aimed to reproduce the native center of rotation every possible time (a high hip center was only accepted in cases of very severe superolateral bone deficit). All the cups were implanted using a conservative circumferential reaming technique, admitting slight medialization in shallow acetabula. Cup inclination was set at 40–45°, with no more than one-third superior undercoverage. Cup anteversion was determined avoiding an anterior overhang (or reducing it to a minimum), aiming for 10–20° of anteversion. The stems were positioned with the aim of achieving an acceptable combined anteversion according to Dorr and Widmer, no offset reduction, adequate canal filling, the most neutral positioning in the coronal and sagittal planes, and leg length equalization, avoiding over-lengthening superior to 3 cm. For all the stems, the surgical planning was performed according to the instructions provided by the manufacturers and the experience of the planner.

### Reliability of the simulations and comparison with post-operative measurements

Fifteen hips with a post-operative CT scan performed for contralateral hip planning and achieving a 2-year excellent clinical outcome with good signs of radiographic osseointegration were selected. In all cases, an anatomical stem was implanted. The first author, blinded to the post-operative component positioning, performed the simulation on the 15 hips. Another simulation was performed by the same author on the same hips after 4 weeks. All the tests were compared to the post-operative component positioning as provided by the CT scan. The outcomes and the reference values were the same as explained below.

### Demographics of the cohort and assessment of the native hip morphology

The demographics of the patients were collected. On the CT scan, the DDH pathology was classified according to Crowe et al. and Hartofilakidis et al. [14]. The native femoral morphology was assessed in the coronal and axial planes [1–3, 15, 16] (Table 1).

**Table 1:** The native hip morphology was described using several CT-based measurements (mean ± standard deviation; median in parentheses): the features that are correlated with the Crowe classification are shown in bold (*LT* lesser trochanter)

Feature of the native hip morphology	Crowe classification				Spearman *ρ* (*p* value)
	I (92 hips)	II (62 hips)	III (37 hips)	IV (9 hips)	
**Center of rotation height (mm)**	17.98 ± 5.20 (18)	22.08 ± 6.42 (21)	28.84 ± 6.24 (30)	43.44 ± 9.62 (42)	0.598 (*p* < 0.0001)
Neck-shaft angle (°)	128.47 ± 13.56 (129.5)	130.43 ± 14.80 (132)	136.11 ± 15.94 (138)	128.22 ± 23.68 (132)	0.167 (*p* = 0.018)
**Femoral offset (mm)**	31.90 ± 8.18 (33)	30.29 ± 7.09 (30)	25.97 ± 6.78 (25)	24.11 ± 9.31 (22)	− 0.295 (*p* < 0.0001)
Acetabular offset (mm)	34.79 ± 4.61 (35)	36.27 ± 5.99 (36)	35.00 ± 6.36 (34)	39.00 ± 4.06 (39)	0.109 (*p* = 0.121)
Axial diameter of acetabular cavity (mm)	46.41 ± 4.73 (46)	46.52 ± 5.22 (46)	45.62 ± 5.29 (46)	39.56 ± 9.10 (44)	− 0.096 (*p* = 0.172)
**Acetabular anteversion (°)**	19.80 ± 7.24 (20)	17.31 ± 9.04 (18)	14.03 ± 9.69 (14)	14.67 ± 10.72 (16)	− 0.243 (*p* = 0.0005)
**Femoral anteversion (°)**	25.55 ± 17.01 (24.5)	38.77 ± 18.23 (40.5)	35.86 ± 19.28 (36)	49.44 ± 20.52 (42)	0.338 (*p* < 0.0001)
**Angle between calcar femorale and posterior condyles (°)**	35.64 ± 17.04 (35)	45.11 ± 17.00 (46.5)	42.70 ± 20.00 (45)	56.38 ± 32.24 (53)	0.214 (*p* = 0.002)
**Leg length discrepancy (mm)**	− 3.04 ± 12.38 (0)	− 11.64 ± 13.00 (− 10)	− 12.43 ± 15.39 (− 15)	− 27.67 ± 20.95 (− 26)	− 0.389 (*p* < 0.0001)
**Mediolateral femoral diameter 2 cm above the LT (mm)**	43.03 ± 6.47 (43)	41.44 ± 6.53 (42)	37.03 ± 6.39 (37)	34.56 ± 4.90 (34)	− 0.346 (*p* < 0.0001)
Mediolateral femoral diameter at LT (mm)	24.24 ± 4.44 (24)	24.16 ± 4.52 (24)	22.51 ± 4.16 (22)	22.11 ± 3.02 (22)	− 0.114 (*p* = 0.105)
Mediolateral femoral diameter at isthmus (mm)	9.50 ± 1.84 (10)	9.92 ± 1.91 (10)	10.68 ± 2.43 (11)	9.67 ± 2.12 (9)	0.143 (*p* = 0.042)
Isthmus position (from LT) (mm)	114.88 ± 19.18 (116)	117.92 ± 12.67 (118)	114.24 ± 17.91 (113)	108.44 ± 10.25 (109)	− 0.066 (*p* = 0.346)
**Canal flare index**	4.67 ± 1.05 (4.5)	4.30 ± 0.91 (4.35)	4.45 ± 4.75 (3.7)	3.76 ± 0.80 (3.8)	− 0.324 (*p* < 0.0001)

### Outcome measurements

In all the simulations, the following measurements were taken (cup measurements were unique to every case; stem measurements were performed for every stem in every case): cup anteversion, stem anteversion, acetabular offset, femoral offset, sagittal and coronal tilt, and canal filling at the mid-third of the stem [1, 11, 17, 18].

When planning, the following five outcomes had to be matched for the stem to be considered acceptable:

- Combined anteversion (according to Dorr et al.) between 25° and 50° and combined anteversion (according to Widmer et al.) < 37° (both the targets had to be matched) [11];
- Global offset (acetabular + femoral offset) loss not inferior to 12% of the native global offset [19];
- Coronal and sagittal stem tilt < 5° [17];
- Canal filling > 80% [18];
- Leg lengthening < 3 cm [20].

### Statistical analysis

The analyses were performed using SPSS 14.0 (SPSS Inc, Chicago, IL, USA). Quantitative data are reported as average values, standard deviations and minimum to maximum ranges. Qualitative data are expressed as frequencies and percentages and were tested using the chi-squared test. The reliability of the simulations was assessed using Fisher’s test (categorical variables). Correlations between parameters were assessed using Kendall, Spearman or Pearson coefficients, depending on the data type. The correlation strength was evaluated according to the current medical literature [21]. Threshold for significance: *p* = 0.05.

## Results

### Demographics and assessment of the native hip morphology

The cohort encompassed 35 males (23.3%) and 115 females (76.7%), with a mean age at the CT scan of 50.8 ± 11 years (range: 19–81). The mean height was 159.8 ± 18.3 cm (range: 128–185), the mean weight was 66.1 ± 15.3 kg (range: 40–112): the average BMI was 25.8 ± 4.9 kg/m^2^ (range: 17.2–39.6). According to the Crowe classification, 92 hips (42%) were graded as I, 62 (32%) as II, 37 (18.5%) as III and 9 (4.5%) as IV. According to the Hartofilakidis classification, 97 hips (48.5%) were classified as A, 59 (29.5%) as B1, 35 (17.5%) as B2, 2 (1%) as C1 and 7 (3.5%) as C2. Both classifications were statistically correlated (Kendall *τ* = 0.956, *p* < 0.0001). The features of the native hip morphology and the correlations with the Crowe classification are provided in Table 1. The center of rotation height progressively increased with the Crowe classification (moderate correlation), as well as the femoral anteversion (fair), the leg length discrepancy (fair) and the angle between the calcar femorale and the tangent to the posterior condyles (poor). The femoral offset (fair correlation), the mediolateral femoral diameter 2 cm above the lesser trochanter (fair), the canal flare index (fair) and the acetabular anteversion (poor) progressively decreased with increasing DDH degree (Crowe classification). The same correlations with the same coefficients were evident when the Hartofilakidis classification was adopted.

### Reliability of the simulations

There was perfect agreement between the simulations and the post-operative CT measurements for combined anteversion, leg lengthening, coronal tilt, sagittal tilt and canal filling. There was no significant difference in offset restoration (Fisher's test, *p* = 0.141). Perfect intra-observer reliability was recorded for all six measured parameters.

### Percentages of the implants that provide adequate reconstruction for each stem design

**Fig. 1:**
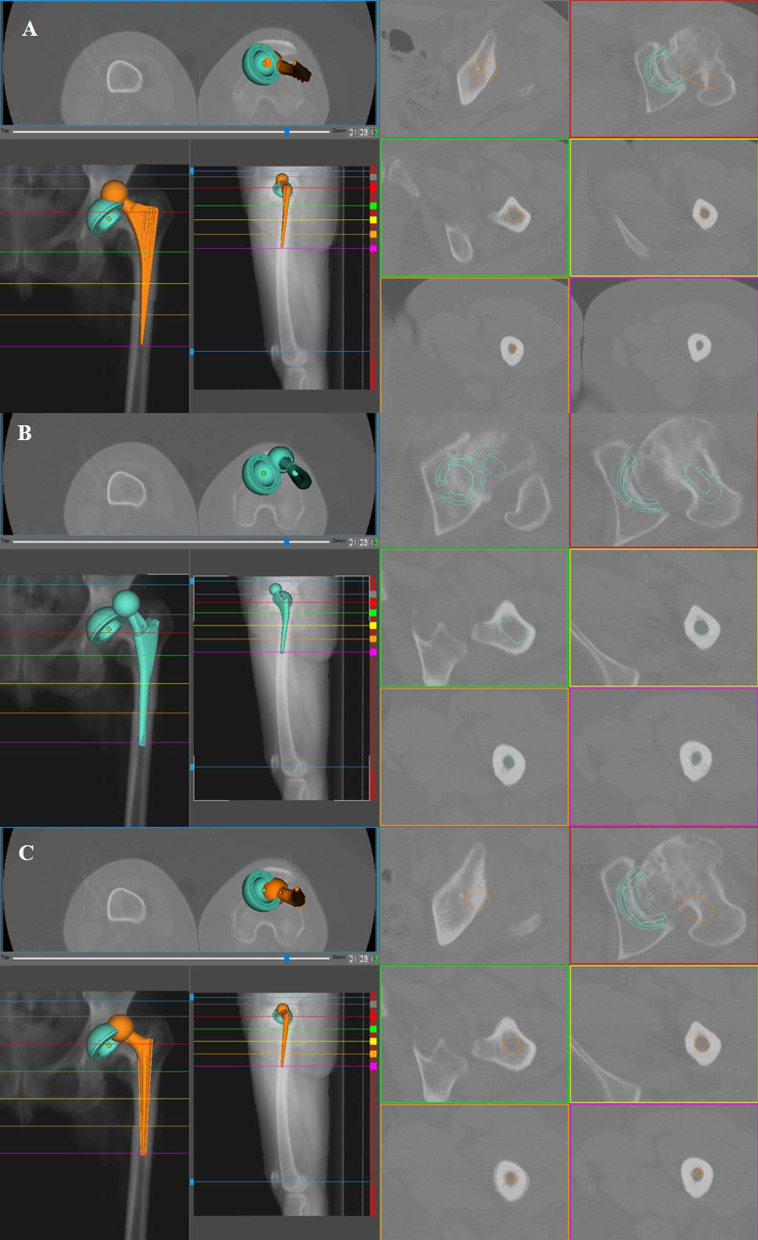
Single wedge (**A**), anatomical (**B**) and conical (**C**) stem simulations were performed in the Crowe II left hip of a 51-year-old female (native femoral anteversion 24°). Single wedge and anatomical stems did not achieve the targeted combined anteversion, whereas the conical stem was deemed acceptable

The stem design providing the highest percentage of adequate hip reconstruction (i.e., all six parameters were matched) was the conical implant (87% of the cases, chi-square test, *p* < 0.0001) (Fig. 1). Out of the 13% of conical implants that did not achieve adequate reconstruction, more than half of those cases did not show good offset restoration (7% of all the implants) (chi-square test, *p* < 0.0001). Single wedge and anatomical stems could not be simulated due to abnormal femoral anatomy in 4.5% and 0.5% of the cases, respectively. Conical implants matched the combined anteversion target in almost all cases; the anatomical stem was the worst performer (Table 2).

**Table 2:** The percentage of acceptable implants for every stem design (every single parameter was evaluated; the last row shows the percentages of acceptable implants that matched all six criteria)

Reconstructive parameter	Acceptable	Stem design			Chi-square test
		Single wedge	Anatomical	Conical	
Combined anteversion	Yes	153 (76.5%)	121 (60.5%)	197 (98.5%)	*p* < 0.0001
	No	47 (22.5%)	79 (39.5%)	3 (1.5%)	
Offset restoration	Yes	192 (96%)	164 (82%)	186 (93%)	*p* < 0.0001
	No	8 (4%)	36 (18%)	14 (7%)	
Coronal tilt	Yes	200 (100%)	200 (100%)	199 (99.5%)	*p* = 0.367
	No	–	–	1 (0.5%)	
Sagittal tilt	Yes	199 (99.5%)	200 (100%)	199 (99.5%)	*p* = 0.605
	No	1 (0.5%)	–	1 (0.5%)	
Canal filling	Yes	199 (99.5%)	177 (88.5%)	198 (99%)	*p* < 0.0001
	No	1 (0.5%)	23 (11.5%)	2 (1%)	
Leg lengthening	Yes	188 (94%)	183 (91.5%)	194 (97%)	*p* = 0.063
	No	12 (6%)	17 (8.5%)	6 (3%)	
All the parameters	Yes	138 (69%)	88 (44%)	174 (87%)	*p* < 0.0001
	No	62 (31%)	112 (56%)	26 (13%)	

### Correlations between the reconstructions provided by the stem designs and the native morphology

**Fig. 2:**
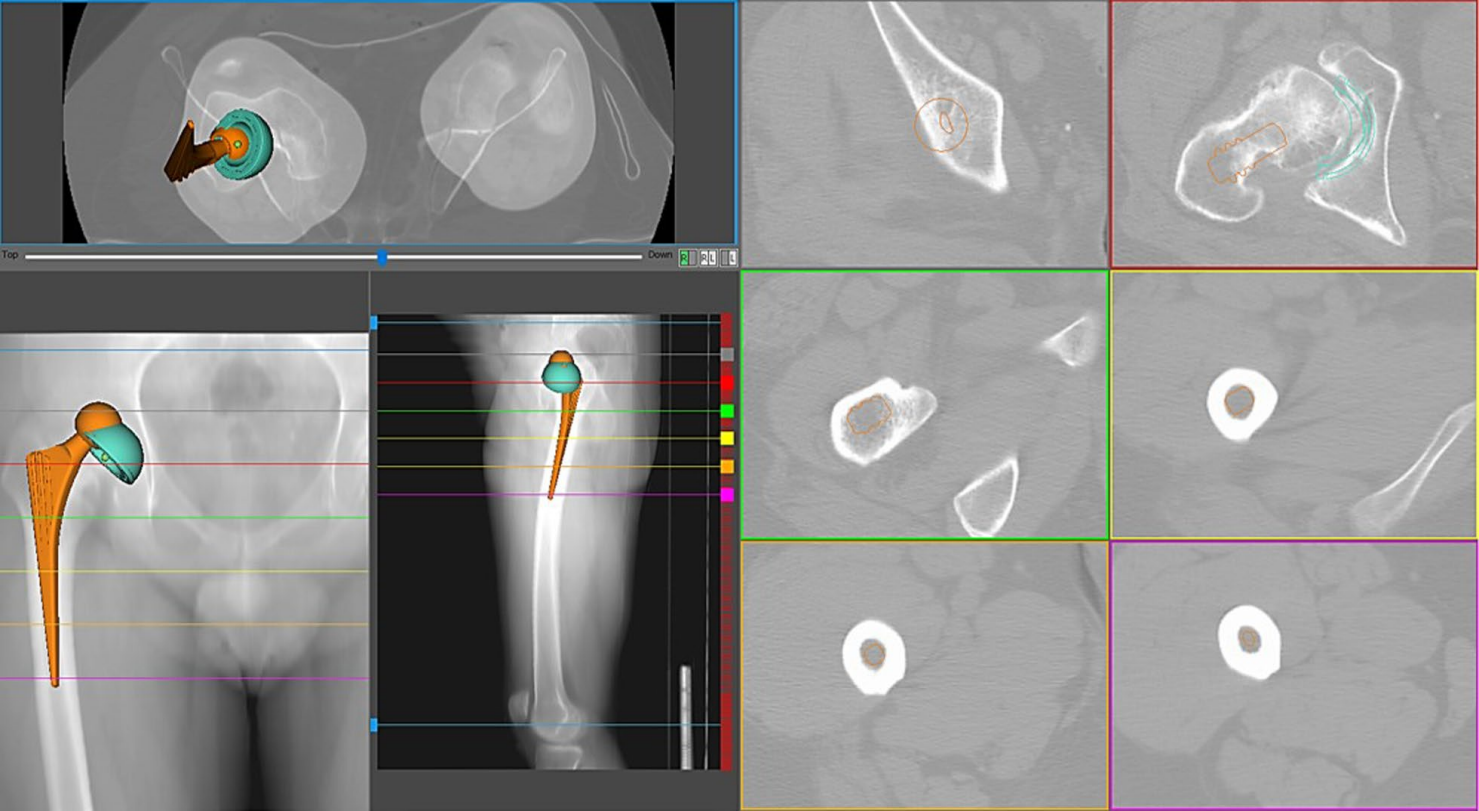
A single wedge stem simulation was performed in the Crowe II right hip of a 41-year-old male (native femoral anteversion 24°). The single wedge stem matched all the reconstructive parameters and achieved an acceptable reconstruction

**Fig. 3:**
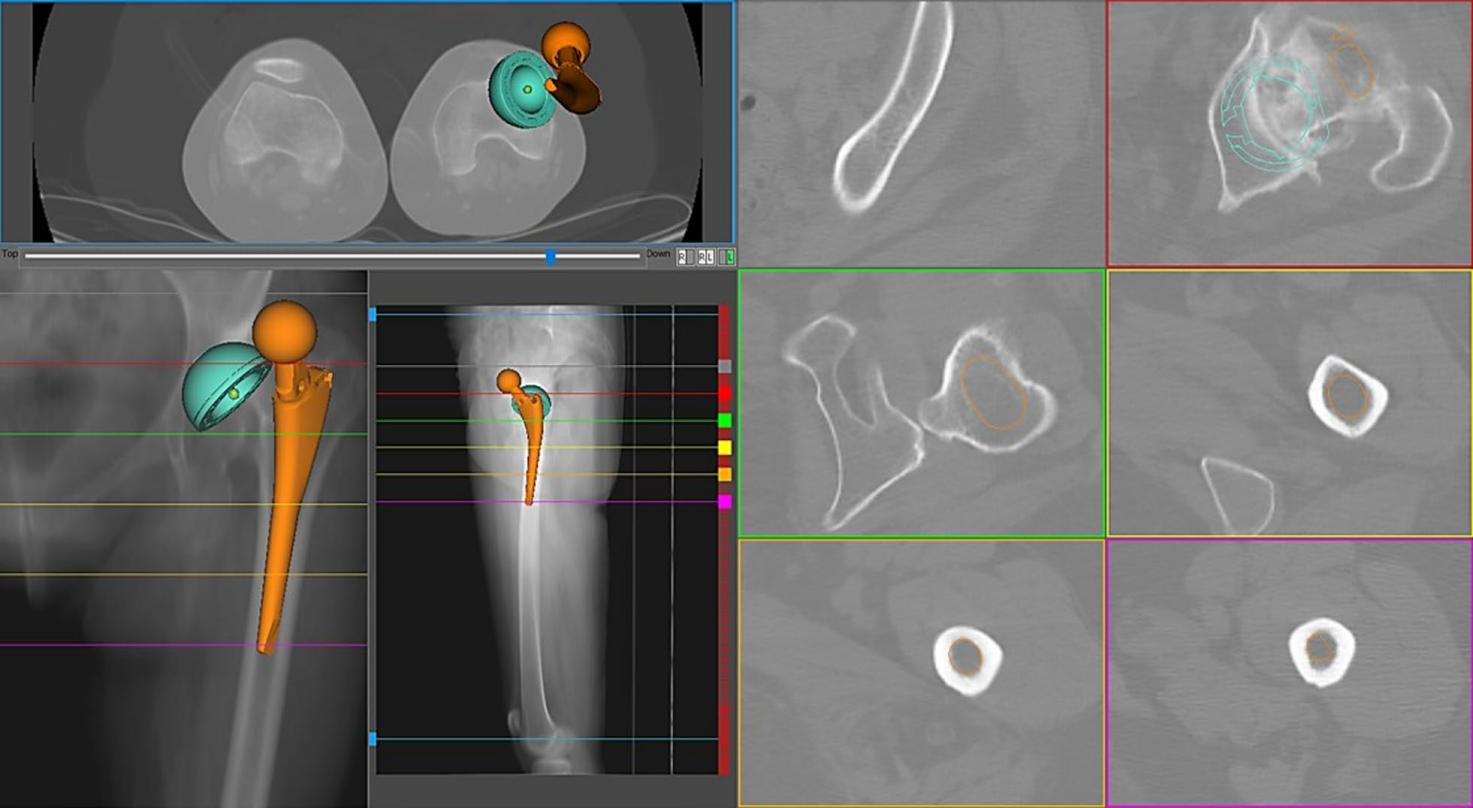
An anatomical stem simulation was performed in the Crowe III dysplastic left hip of a 46-year-old female (native femoral anteversion 60°). Even with a cup anteversion of 16°, combined anteversion was beyond the limits (Dorr 70°, Widmer 54°), and the reconstruction was unacceptable

The reconstructions provided by the stem designs were significantly correlated with the Crowe classification. For Crowe I and II hips, the single wedge implant provided an acceptable reconstruction in 70.8% of the cases, the anatomical stem in 44.8% and the conical device in 91.6% (Pearson’s chi-square test, *p* < 0.0001) (Fig. 2). For Crowe III and IV hips, the same designs achieved percentages of acceptability of 63%, 41.3% and 71.7%, respectively (Pearson’s chi-square test, *p* < 0.0094) (Fig. 3). Similar percentages were achieved with the Hartofilakidis classification, apart from C hips (the single wedge and conical stem performed equally well, *p* 0.837).

The acceptability of the single wedge stems was mainly correlated with combined anteversion (Pearson’s *r* = 0.827, *p* < 0.0001: very strong correlation), leg lengthening < 3 cm (Pearson’s *r* = 0.377, *p* < 0.0001: fair correlation) and offset reconstruction (Pearson’s *r* = 0.308, *p* < 0.0001: fair correlation) (Table 3). The acceptability of the anatomical stems was mainly correlated to combined anteversion (Pearson’s *r = * 0.716, *p* < 0.0001: moderate correlation), offset reconstruction (Pearson’s *r =* 0.415, *p* < 0.0001: fair correlation), canal filling (Pearson’s *r =* 0.320, *p* < 0.0001: fair correlation) and leg lengthening < 3 cm (Pearson’s *r =* 0.270, *p* 0.0001: fair correlation). For the conical stems, the acceptability was correlated to offset reconstruction (Pearson’s *r =* 0.710, *p* < 0.0001: moderate correlation), leg lengthening < 3 cm (Pearson’s *r =* 0.455, *p* < 0.0001: fair correlation), combined anteversion (Pearson’s *r =* 0.319, *p* < 0.0001: fair correlation) and canal filling (Pearson’s *r =* 0.26, *p* < 0.0002: fair correlation).

**Table 3:** The correlations between the reconstructive parameters and the native morphology for each stem design (only fair correlations are reported)

Reconstructive parameter	Stem design		
	Single wedge	Anatomical	Conical
Combined anteversion	Femoral anteversion (Pearson’s *r =* − 0.295, *p* < 0.0001: fair inverse correlation)	Femoral anteversion (Pearson’s *r =* − 0.274, *p* < 0.0001: fair inverse correlation)	No correlations
Offset reconstruction	No correlations	No correlations	Offset (Pearson’s *r =* − 0.257, *p* 0.0002: fair inverse correlation)
Coronal and sagittal tilt	No correlations	No correlations	No correlations
Canal filling	No correlations	No correlations	No correlations
Leg lengthening	Center of rotation height (Pearson’s *r =* − 0.308, *p* < 0.0001: fair inverse correlation)	Center of rotation height (Pearson’s *r =* − 0.361, *p* < 0.0001: fair inverse correlation)	Center of rotation height (Pearson’s *r =* − 0.450, *p* < 0.0001: fair inverse correlation)
	Neck-shaft angle (Pearson’s *r =* 0.270, *p* 0.0001: fair correlation)		
	Calcar femorale (Pearson’s *r* = − 0.266, *p* 0.0001: fair inverse correlation)	Center of rotation height (Pearson’s *r* = − 0.361, *p* < 0.0001: fair inverse correlation)	Center of rotation height (Pearson’s *r =* − 0.450, *p* < 0.0001: fair inverse correlation)
Acceptability	Femoral anteversion (Pearson’s *r =* − 0.334, *p* < 0.0001: fair inverse correlation)	Femoral anteversion (Pearson’s *r =* − 0.253, *p* 0.0003: fair inverse correlation)	No correlations
	Calcar femorale (Pearson’s *r =* − 0.240, *p* 0.0006: poor correlation)		

In general, stem anteversion was correlated with femoral anteversion (Pearson’s *r =* 0.380, *p* < 0.0001: fair correlation), the angle between the calcar femorale and the posterior femoral condyles (Pearson’s *r =* 0.284, *p* < 0.0001: fair correlation), and the mediolateral femoral diameter at isthmus (Pearson’s *r =* − 0.133, *p* 0.0012: poor correlation). When the femoral anteversion was ≥ 25°, combined anteversion acceptability was 99.2% for the conical stem, 71.4% for the single wedge stem and 51.6% for the anatomical stem (Pearson’s chi-square test, *p* < 0.0001) (Table 4). The same trend with femoral anteversion was observed for acceptable reconstructions based on all six criteria. For offset reconstruction, all the stem designs provided adequate percentages (single wedge 96.9%; anatomical 84.6%; conical 90.8%) of offset restoration when the native offset was normal (37 ± 4 mm), with no significant differences between the stems (Pearson’s chi-square test, *p* = 0.056) [19]. When the native offset was beyond the normal limits, the single wedge stem achieved the best performance (95.6%, Pearson’s chi-square test, *p* < 0.0001), followed by the conical (94.1%) and anatomical (80.7%) stems.

**Table 4:** The acceptable combined anteversions and acceptable reconstructions (all six criteria were matched) for every stem design stratified according to two classes of femoral anteversion; note that the differences between the groups were significant

Femoral anteversion	Stem design						Pearson’s chi-square test (*p* value)
	Single wedge		Anatomical		Conical		
	Acceptable combined anteversion		Acceptable combined anteversion		Acceptable combined anteversion		
	Yes	No	Yes	No	Yes	No	
< 25° (74 cases)	63 (85.1%)	11 (14.9%)	52 (70.3%)	23 (29.7%)	72 (97.3%)	2 (2.7%)	< 0.0007
≥ 25° (126 cases)	90 (71.4%)	36 (28.6%)	65 (51.6%)	61 (48.4%)	125 (99.2%)	1 (0.8%)	< 0.0001

	Acceptable reconstruction (all parameters)		Acceptable reconstruction (all parameters)		Acceptable reconstruction (all parameters)		Pearson’s chi-square test (*p* value)
	Yes	No	Yes	No	Yes	No	
< 25° (74 cases)	58 (78.4%)	16 (21.6%)	44 (59.5%)	30 (40.5%)	63 (85.1%)	11 (14.9%)	0.0096
≥ 25° (126 cases)	80 (63.5%)	46 (37.5%)	43 (34.1%)	83 (65.9%)	111 (88.1%)	15 (11.9%)	< 0.0001

## Discussion

Stem choice in DDH is still a matter of debate [6]. There are no available guidelines, only generic recommendations for conical implants in cases with small femurs, high anteversions and abnormal anatomies [4–6]. The study showed that conical stems provided the best percentage of acceptable implants in DDH. Stem choice was mainly dictated by combined anteversion; in particular, stem anteversion was correlated with the native femoral anteversion, the calcar femorale and the mediolateral diameter of the femur. Conical stems adequately reconstructed the hip biomechanics, even in the case of high femoral anteversion, ≥ 25°.

The simulation of three different stem designs in the same 200 randomly selected hips provided a valuable tool to define appropriate indications and thresholds for stem choice in DDH. However, this study has some methodological limits: the single-surgeon planning technique, the variability of femoral anteversion measurements and the small cohort of high-degree dysplasia. On the other hand, as shown by the description of the native dysplastic morphology, a wide range of femoral variations and axial anatomies were included in the study. Some exceptional cases were probably excluded or poorly represented; however, THA simulations of outliers are beyond the scope of the study. A single stem simulation per stem group was provided, according to the available software library, the collaboration of manufacturers and the simulator’s proficiency with the specific stem. The simulated reconstructions provided by each stem should be considered indicative of any other stem belonging to the same group: minor differences due to the variability of the proximal biomechanics of other similar stems do not invalidate the general conclusions. Modular stems were intentionally excluded: these devices are valid alternatives in complex anatomies but at the cost of possible adjunctive failures, especially in young and active males [20].

From a practical perspective, the study showed that single wedge biomechanical reconstructions in DDH were mainly influenced by the combined anteversion, and thus benefited from lower native femoral anteversion and a lower calcar femorale angle. Similarly, anatomical stem acceptability was impacted by the combined anteversion and consequently was favored by lower native femoral anteversion. Single wedge stems matched the correct combined anteversion in more cases than anatomical stems, confirming that tapered stems may allow a larger anteversion adjustment than metaphyseal fitting stems [5]. However, single wedge stems could not address as many femoral deformities as conical implants, which matched the correct combined anteversion in almost all cases (> 97%) regardless of the native femoral anteversion, thanks to the free adjustment of the version [6]. Conversely, most of the conical stems that did not fulfill the criteria of good reconstruction in DDH failed due to inadequate offset restoration (7%), highlighting a well-known drawback of these implants [6]. Thus, considering the good percentage of hip reconstruction, with only a small percentage of cases showing non-optimal offset restoration, it could be concluded that conical stems should be recommended for most (all) dysplastic hips. However, considering that conical stems have a diaphyseal fitting design that violates the distal bone stock, and offset reconstruction may be impaired in low-degree DDH, the routine adoption of conical stems in DDH cases does not seem warranted [6] (Table 1).

Thus, we could assume that the pre-operative planning of THA in DDH should play a pivotal role in discriminating between stems. However, the sole coronal images with Crowe and Hartofilakidis classifications are not informative enough to allow a mindful choice in every case, as bidimensional classifications have only a fair correlation with axial femoral parameters, as demonstrated by the standard deviations and the medians of the four Crowe cohorts (Table 1). As adequate reconstruction of hip biomechanics is mainly based on appropriate combined anteversion and stem anteversion, which depends on the mediolateral diameter at the isthmus, the calcar femorale and the femoral anteversion, axial imaging seems to be of help when choosing the most appropriate stem.

Thus, it is difficult to provide a simple femoral classification for stem choice in DDH, as the native femoral morphology develops along the coronal and the axial planes simultaneously. A pre-operative CT scan with dedicated protocols may allow comprehensive planning (with no more concerns about radiation exposure) [22]. However, as a rule of thumb, tapered stems can provide adequate reconstruction in cases with femoral anteversion < 25° (thus, in most low-degree DDH), and have the advantage of respecting the diaphyseal bone stock and producing the best offset reconstruction. Conical stems should be implanted in cases with femoral anteversion ≥ 25°, but also in cases with small femurs and abnormal anatomies regardless of the magnitude of femoral anteversion.

## Data Availability

The datasets used and/or analyzed during the current study are available from the corresponding author on reasonable request.
